# Prion-Associated Neurodegeneration Causes Both Endoplasmic Reticulum Stress and Proteasome Impairment in a Murine Model of Spontaneous Disease

**DOI:** 10.3390/ijms22010465

**Published:** 2021-01-05

**Authors:** Alicia Otero, Marina Betancor, Hasier Eraña, Natalia Fernández Borges, José J. Lucas, Juan José Badiola, Joaquín Castilla, Rosa Bolea

**Affiliations:** 1 Centro de Encefalopatías y Enfermedades Transmisibles Emergentes, Universidad de Zaragoza IA2 IIS Aragón, 50013 Zaragoza, Spain; aliciaogar@unizar.es (A.O.); mbetancorcaro@gmail.com (M.B.); badiola@unizar.es (J.J.B.); 2 ATLAS Molecular Pharma S.L., Parque tecnológico de Bizkaia, 48160 Derio, Spain; herana.atlas@cicbiogune.es; 3 Center for Cooperative Research in Biosciences (CIC bioGUNE) Basque Research and Technology Alliance (BRTA), Bizkaia Technology Park, 48160 Derio, Spain; nataliafernandezb@hotmail.com (N.F.B.); castilla@joaquincastilla.com (J.C.); 4 Centro de Biología Molecular ‘Severo Ochoa’ (CBMSO) CSIC/UAM, 28049 Madrid, Spain; jjlucas@cbm.csic.es; 5 Networking Research Center on Neurodegenerative Diseases (CIBERNED), Instituto de Salud Carlos III, 28031 Madrid, Spain; 6 IKERBasque Basque Foundation for Science, 48009 Bilbao, Spain

**Keywords:** ER stress, endoplasmic reticulum, UPS impairment, proteasome, prions

## Abstract

Prion diseases are a group of neurodegenerative disorders that can be spontaneous, familial or acquired by infection. The conversion of the prion protein PrP^C^ to its abnormal and misfolded isoform PrP^Sc^ is the main event in the pathogenesis of prion diseases of all origins. In spontaneous prion diseases, the mechanisms that trigger the formation of PrP^Sc^ in the central nervous system remain unknown. Several reports have demonstrated that the accumulation of PrP^Sc^ can induce endoplasmic reticulum (ER) stress and proteasome impairment from the early stages of the prion disease. Both mechanisms lead to an increment of PrP aggregates in the secretory pathway, which could explain the pathogenesis of spontaneous prion diseases. Here, we investigate the role of ER stress and proteasome impairment during prion disorders in a murine model of spontaneous prion disease (TgVole) co-expressing the Ub^G76V^-GFP reporter, which allows measuring the proteasome activity in vivo. Spontaneously prion-affected mice showed a significantly higher accumulation of the PKR-like ER kinase (PERK), the ER chaperone binding immunoglobulin protein (BiP/Grp78), the ER protein disulfide isomerase (PDI) and the Ub^G76V^-GFP reporter than age-matched controls in certain brain areas. The upregulation of PERK, BiP, PDI and ubiquitin was detected from the preclinical stage of the disease, indicating that ER stress and proteasome impairment begin at early stages of the spontaneous disease. Strong correlations were found between the deposition of these markers and neuropathological markers of prion disease in both preclinical and clinical mice. Our results suggest that both ER stress and proteasome impairment occur during the pathogenesis of spontaneous prion diseases.

## 1. Introduction

Prion diseases or transmissible spongiform encephalopathies (TSE) are a group of fatal neurodegenerative disorders produced by the accumulation of the pathological prion protein (PrP^Sc^) in the central nervous system (CNS) of affected individuals [1]. TSE can have a spontaneous, familial or infectious origin, but all prion-related disorders share a main pathogenic event: the conversion of the physiological cellular prion protein (PrP^C^) into its misfolded, abnormal isoform PrP^Sc^ [1,2].

Even though the pathogenic mechanisms underlying prion diseases are not completely understood, it has been suggested that ER stress, induced by PrP^Sc^, could play an important role in prion-associated neurodegeneration [3,4,5,6]. PrP^Sc^ accumulation has been reported to initially disturb ER calcium homeostasis [5,7] and to trigger the Unfolded Protein Response (UPR), a pro-survival mechanism which can be activated by the persistent accumulation of misfolded proteins in the ER producing ER stress [8,9]. PRKR-like endoplasmic reticulum kinase (PERK) is one of the major sensor proteins initiating the UPR, selectively interacting with misfolded proteins [10]. In nonstress conditions, PERK is bound to the binding immunoglobulin protein (BiP/Grp78), the principal ER chaperone, which has been suggested to be the key component for the detection of ER stress [11,12]. When ER stress is detected, BiP dissociates from PERK, producing its oligomerization and autophosphorylation and initiating the UPR to restore the equilibrium in the ER [13,14]. The UPR results in the upregulation of ER chaperones and foldases, the decrease of protein synthesis and the induction of protein elimination through ER-associated degradation (ERAD), a cellular pathway that involves the retro-translocation of misfolded proteins into the cytosol and their degradation by the ubiquitin-proteasome system (UPS) [8,15,16]. Although the role of the UPR in the pathogenesis of prion diseases is not completely understood, the upregulation of certain ER chaperones involved in this pathway, including BiP, and protein disulfide isomerases such as PDIA1 (PDI) have been demonstrated in prion diseases [6,17,18,19]. BiP expression has been reported to be increased in postmortem brain samples of variant Creutzfeldt Jakob disease (vCJD) and sporadic CJD (sCJD) patients [7]. On the other hand, no activation of the PERK pathway has been detected in any form of human prion disease [20].

PDI is a folding enzyme present in the ER lumen at high concentrations [21]. This protein plays a key role in oxidative folding, catalyzing the formation and rearrangement of disulfide bonds in proteins [22,23] but also acts as a molecular chaperone, promoting a correct folding and preventing aggregation of misfolded proteins [22,24,25,26]. The upregulation of proteins of the PDI family in the brain of prion-affected individuals has been described in both sCJD and vCJD patients [7,18,27] and in prion-infected rodents [6,17]. The overexpression of these proteins was suggested to be a defensive response against the presence of PrP^Sc^ [7,17,18]. In vitro studies using cell cultures demonstrated that PDI family member Grp58 interacts with PrP, acting as a neuroprotective factor [17]. However, it was also shown that PDI could have a proapoptotic effect in models of protein misfolding diseases, leading to a cascade of caspases and apoptotic cell death [28]. Nevertheless, Wang et al., 2012 demonstrated that the role of PDI during the course of prion disease is complex, exerting a protective activity against PrP^Sc^ at the early stages but inducing apoptosis at the terminal point of the disease.

When the cell experiences ER stress, protein elimination through the UPS is improved [16]. Misfolded and unnecessary proteins to be eliminated via the UPS are tagged by multiple ubiquitin molecules and subsequently degraded by the 26S proteasome [29]. However, it has been suggested that, during the pathogenesis of prion diseases, an impairment of this natural defensive mechanism may occur. Histopathological studies showed the accumulation of ubiquitinated aggregates in the brain in human prion diseases [30,31]. Subsequent experiments, using transgenic mice, have corroborated the aggregation of these ubiquitinated conjugates in the brain of prion-infected animals [32,33,34], and in vitro studies have shown that PrP^Sc^ is able to inhibit the proteolytic β subunits of the proteasome, decreasing its activity [33]. All these results indicate that the deterioration of the UPS function may play an important role in the pathogenesis of neurodegeneration in prion diseases.

Both ER stress and UPS impairment, induced by PrP^Sc^, can be correlated, since chronic ER stress leads to an accumulation of nontranslocated PrP in the cytosol [35,36] that could deteriorate the proteasome activity [33]. In vitro and in vivo studies have shown that cells under chronic ER stress fail to clear efficiently the aberrant proteins, which accumulate and cause an impairment of the UPS [37]. In addition, both ER stress and proteasome activity inhibition lead to an increase of insoluble PrP aggregates in the secretory pathway and cause a significant accumulation of PrP^Sc^ in persistently prion-infected cells, suggesting that, therefore, these mechanisms could be involved in the de novo formation of prions [38].

However, other studies failed to detect ER stress or proteasomal malfunction in transgenic models of familial prion diseases [39]. In the present study, we investigated the role of ER stress and UPS impairment in a mouse model for sporadic (putatively spontaneous) prion diseases: the TgVole mouse model. These mice overexpress bank vole I109 PrP, which always leads to the development of a spontaneous prion disease. This spontaneous prion disease shows identical severity and clinical signs (mild kyphosis and rapidly progressing ataxia) even in different cohorts of animals [40,41]. Moreover, it has also been demonstrated in previous experiments that TgVole mice develop this spontaneous prion disease with 100% attack rates even when mice express amino acid substitutions associated with resistance to prions [41]. Therefore, we consider that the TgVole line is a reliable model for time-course studies of spontaneous prion diseases.

Since no infectious or genetic cause has been found to explain the origin of sporadic prion diseases, it is considered that sporadic prion diseases appear spontaneously in the host, but the exact origin of sporadic TSE is yet not known. Therefore, it is not known whether they really occur spontaneously or there are any other underlying mechanisms that trigger these diseases, and that is why they are considered “putatively” or “supposedly” spontaneous. Several hypotheses have been proposed to explain the molecular basis of spontaneous prion diseases [40]: (a) an aleatory misfolding process that generates PrP^Sc^ molecules from PrP^C^, (b) the presence of somatic mutations in the PrP gene in a reduced number of cells that generate mutant PrP molecules that afterwards template the formation of PrP^C^ into PrP^Sc^ and (c) discordances between mRNA sequences and DNA sequences.

To evaluate the functional status of the UPS during this spontaneous prion disorder, TgVole mice were crossed with Ub^G76V^-GFP/1 reporter mice (hereafter referred to as TgU1 mice). The deposition of the Ub^G76V^-GFP reporter indicates UPS dysfunction [42]. We also studied the accumulation of PERK, BiP and PDI in the brains of these mice, revealing those cells undergoing ER stress in this hybrid transgenic model. Our results show that both ER stress and UPS dysfunction are significantly incremented in certain brain areas in the groups of preclinical and clinical mice. In addition, the accumulation of protein aggregates was more intense in specific cell populations.

## 2. Results

### 2.1. Mice Affected by the Spontaneous Prion Disease Show Increased Accumulation of ER Stress Markers in Certain Brain Areas

This study was performed using hybrid transgenic mouse models of spontaneous disease, euthanized at different ages: TgU1^+^/TgVole^+^ mice that developed the spontaneous prion disorder (hereafter referred to as clinical TgU1^+^/TgVole^+^ mice, euthanized at ~180–200 days of age) and TgU1^+^/TgVole^+^ mice that were euthanized at ~60 days of age before the onset of the disease (hereafter referred to as preclinical TgU1^+^/TgVole^+^ mice). The accumulation of the ER stress markers BiP (Grp78), PERK and PDI was analyzed in these animals and in two groups of age-matched TgU1^+^/TgVole^−^ mice that were used as controls (hereafter referred to as clinical TgU1^+^/TgVole^−^ controls and preclinical TgU1^+^/TgVole^−^ controls, respectively). TgU1^+^/TgVole^−^ mice do not develop a spontaneous prion disease.

Before analyzing the data, the deposition of these proteins was evaluated in several TgU1^−^/TgVole^+^ mice to know whether the expression of the Ub-GFP reporter affects the deposition of these ER stress markers. It was found that TgU1^−^/TgVole^+^ mice showed an identical PERK, BiP and PDI accumulation pattern to age-matched TgU1^+^/TgVole^+^ animals in terms of the morphology of the deposits, intensity and brain distribution of immunostaining, suggesting that the expression of Ub^G76V^-GFP had no apparent effect on the accumulation of ER stress markers (Supplementary Figure S1).

**Figure 1:**
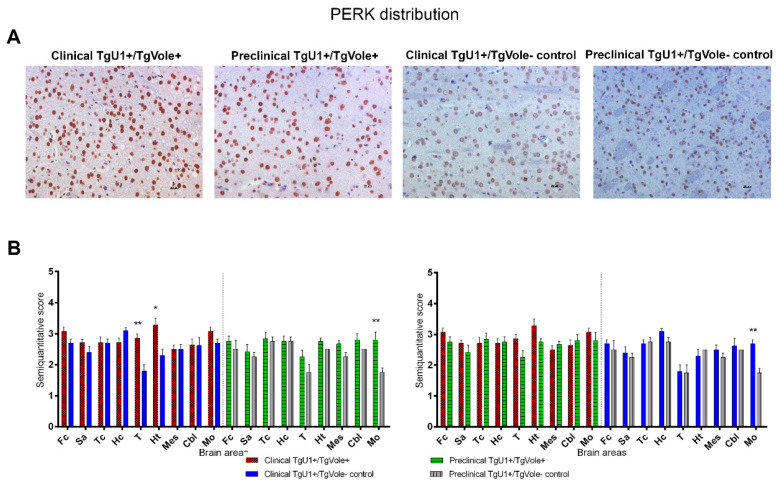
PRKR-like endoplasmic reticulum kinase (PERK) upregulation in the thalamic area of clinical TgU1^+^/TgVole^+^ mice. (**A**) Nuclear staining was observed for PERK in numerous cells of all groups of mice. The number of immunopositive cells was higher and immunostaining was stronger in TgU1^+^/TgVole^+^ mice, especially in the thalamus and hypothalamus. Images correspond to the hypothalamic area in all mice. (**B**) PERK distribution in the brains of clinical and preclinical TgU1^+^/TgVole^+^ mice and their age-matched controls. PERK immunostaining was analyzed using a semiquantitative scale from 0 (lack of immunostaining) to 5 (very intense immunostaining) in nine different brain areas: frontal cortex (Fc), septal area (Sa), cortex at the level of the thalamus (Tc), hippocampus (Hc), thalamus (T), hypothalamus (Ht), mesencephalon (Mes), cerebellum (Cbl) and medulla oblongata (Mo). The number of animals studied was the following: clinical TgU1^+^/TgVole^+^ *n* = 6 (4 female, 2 male), clinical TgU1^+^/TgVole^−^ controls *n* = 8 (6 female, 2 male), preclinical TgU1^+^/TgVole^+^ *n* = 5 (2 female, 3 male) and preclinical TgU1^+^/TgVole^−^ controls *n* = 5 (2 female, 3 male). Comparison of the PERK immunolabeling revealed significant differences between clinical TgU1^+^/TgVole^+^ mice and clinical TgU1^+^/TgVole^−^ controls and between preclinical TgU1^+^/TgVole^+^ mice and preclinical TgU1^+^/TgVole^−^ controls in different brain areas. (* *p* < 0.05, ** *p* < 0.01, Mann-Whitney U test).

PERK staining was observed in cellular nuclei in all groups of mice, an immunohistochemical pattern described for this protein in other studies [43] (Figure 1A). Clinical TgU1^+^/TgVole^+^ mice showed an upregulation of PERK in the thalamus and hypothalamus compared to their TgU1^+^/TgVole^−^ controls, which presented faint PERK immunolabeling in these regions. Preclinical TgU1^+^/TgVole^−^ controls accumulated low levels of this protein in the medulla oblongata, which led to significant differences in deposition when compared with preclinical TgU1^+^/TgVole^+^ mice and clinical TgU1^+^/TgVole^−^ controls (Figure 1B).

**Figure 2:**
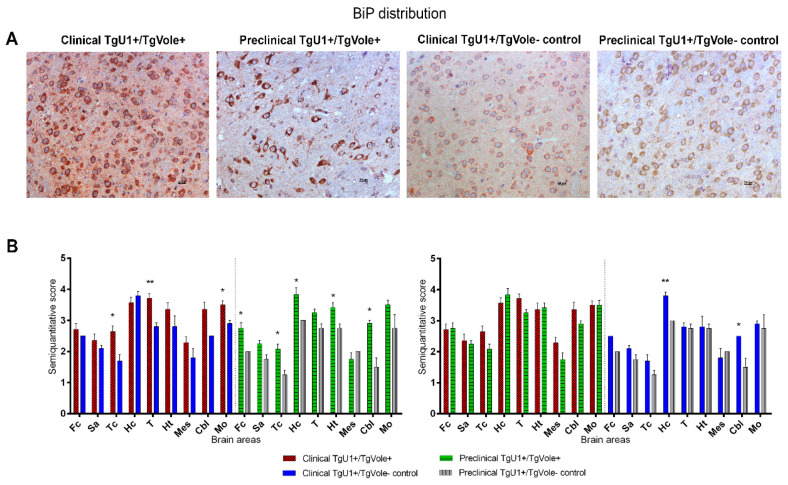
Binding immunoglobulin protein (BiP) expression levels are higher in clinical and preclinical TgU1^+^/TgVole^+^ mice than in healthy TgU1^+^/TgVole^-^ controls in certain brain areas. (**A**) All groups of mice presented uniform cytoplasmic labeling of BiP in numerous neurons and a diffuse staining in the neuropil. Immunostaining intensity, rather than the number of positive cells, was higher in both clinical and preclinical TgU1^+^/TgVole^+^ compared to their age-matched controls. (**B**) BiP distribution in the brains of clinical and preclinical TgU1^+^/TgVole^+^ mice and their age-matched controls. BiP immunostaining was analyzed using a semiquantitative scale from 0 (lack of immunostaining) to 5 (very intense immunostaining) in nine different brain areas: frontal cortex (Fc), septal area (Sa), cortex at the level of the thalamus (Tc), hippocampus (Hc), thalamus (T), hypothalamus (Ht), mesencephalon (Mes), cerebellum (Cbl) and medulla oblongata (Mo). The number of animals included in each group was: clinical TgU1^+^/TgVole^+^ *n* = 6 (4 female, 2 male), clinical TgU1^+^/TgVole^−^ controls *n* = 8 (6 female, 2 male), preclinical TgU1^+^/TgVole^+^ *n* = 5 (2 female, 3 male) and preclinical TgU1^+^/TgVole^−^ controls *n* = 5 (2 female, 3 male). Comparison of the BiP immunolabeling revealed significant differences between clinical TgU1^+^/TgVole^+^ mice and clinical TgU1^+^/TgVole^−^ controls and between preclinical TgU1^+^/TgVole^+^ mice and preclinical TgU1^+^/TgVole^−^ controls in different brain areas. No differences were observed between clinical and preclinical TgU1^+^/TgVole^+^ mice. (* *p* < 0.05, ** *p* < 0.01, Mann-Whitney U test).

BiP deposition was characterized by intense and uniform cytoplasmic immunostaining affecting numerous cells and granular diffuse immunolabeling in the neuropil in all groups of mice (Figure 2A). Clinical TgU1^+^/TgVole^+^ and preclinical TgU1^+^/TgVole^+^ mice showed a statistically significant upregulation of BiP in certain brain areas compared to their age-matched controls. The thalamus was the brain region showing the most intense BiP accumulation in clinical TgU1^+^/TgVole^+^ mice, significantly higher than that observed in clinical TgU1^+^/TgVole^−^ controls. Differences in immunolabeling were also observed in the cortex at the level of the thalamus and medulla oblongata between these two groups of mice. Preclinical TgU1^+^/TgVole^+^ mice showed BiP upregulation compared to their controls in certain brain areas (frontal cortex, cortex at the level of the thalamus, hippocampus, hypothalamus and cerebellum). No differences were observed, however, between BiP deposition of the clinical TgU1^+^/TgVole^+^ and preclinical TgU1^+^/TgVole^+^ mice (Figure 2B).

**Figure 3:**
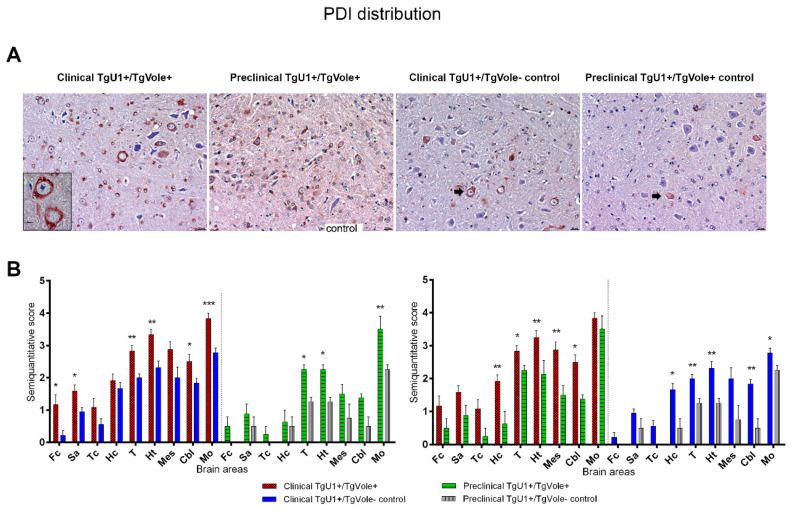
Protein disulfide isomerase (PDI) accumulation is more intense in clinical TgU1^+^/TgVole^+^ mice than in preclinical TgU1^+^/TgVole^+^ mice and healthy TgU1^+^/TgVole^−^ controls in all brain areas. (**A**) A strong intraneuronal PDI labeling was observed in the gigantocellular reticular nucleus of the medulla oblongata of clinical TgU1^+^/TgVole^+^ mice. Intraneuronal PDI immunolabeling was also observed in the other groups of mice, but the intensity of the immunostaining and the number of immunopositive cells were reduced compared with clinical TgU1^+^/TgVole^+^ mice (arrows). Insert picture contains two neurons showing a strong accumulation of PDI. (**B**) PDI distribution in the brains of clinical and preclinical TgU1^+^/TgVole^+^ mice and their age-matched controls. PDI immunolabeling was semiquantitatively analyzed and scored on a scale of 0 (absence of immunolabeling) to 5 (very intense immunolabeling) in nine brain areas. Each group included the following number of animals: clinical TgU1^+^/TgVole^+^ *n* = 6 (4 female, 2 male), clinical TgU1^+^/TgVole^−^ controls *n* = 8 (6 female, 2 male), preclinical TgU1^+^/TgVole^+^ *n* = 5 (2 female, 3 male) and preclinical TgU1^+^/TgVole^−^ controls *n* = 5 (2 female, 3 male). Comparison of the PDI immunolabeling profiles revealed significant differences between the group of clinical TgU1^+^/TgVole^+^ mice and clinical TgU1^+^/TgVole^−^ controls in certain brain areas and between preclinical TgU1^+^/TgVole^+^ mice and preclinical TgU1^+^/TgVole^−^ controls. Significant differences in PDI immunostaining were also noticed between clinical and preclinical TgU1^+^/TgVole^+^ mice and between clinical and preclinical TgU1^+^/TgVole^−^ controls. (* *p* < 0.05, ** *p <* 0.01, *** p < 0.001, Mann-Whitney U test).

PDI accumulation was more intense in clinical TgU1^+^/TgVole^+^ mice in all brain areas. These animals showed a very intense intraneuronal PDI immunolabeling, affecting numerous cells in certain brain regions. PDI-positive immunostaining was found in all groups of mice. However, the PDI accumulation in clinical TgU1^+^/TgVole^−^ controls and preclinical TgU1^+^/TgVole^−^ controls was, in general, weaker and was detected in a smaller number of cells than in clinical TgU1^+^/TgVole^+^ or preclinical TgU1^+^/TgVole^+^ mice. Dark-stained granular aggregates were observed within the cytoplasm of the neurons of all groups of mice studied. This pattern of intracellular immunolabeling, characteristic of ER associated proteins [17,27], was, in general, more evident in clusters of large neurons, such as the deep nuclei of the cerebellum and the gigantocellular reticular nucleus of the medulla oblongata (Figure 3A). A significantly higher accumulation of PDI was demonstrated in the group of clinical TgU1^+^/TgVole^+^ animals in certain brain areas (frontal cortex, septal area, thalamus, hypothalamus and medulla oblongata (*p* < 0.05) when compared with clinical TgU1^+^/TgVole^−^ controls. The medulla oblongata was the region presenting the most intense PDI accumulation in all groups of mice, regardless of age or genotype. Preclinical TgU1^+^/TgVole^+^ mice showed a significantly higher PDI deposition than preclinical TgU1^+^/TgVole^−^ controls in the thalamus, hypothalamus and medulla oblongata, indicating that the upregulation of this protein starts in the preclinical stage of the spontaneous disease. However, the PDI deposition was significantly incremented in clinical TgU1^+^/TgVole^+^ mice compared to preclinical animals, suggesting that the PDI levels increase during the course of the disease. Significant differences between clinical TgU1^+^/TgVole^−^ and preclinical TgU1^+^/TgVole^−^ controls suggest that aging causes an increase in PDI accumulation (Figure 3B).

### 2.2. Mice Affected by the Spontaneous Prion Disease Show Ub^G76V^-GFP accumulation in Brain Areas with Prion-Associated Neuropathology

**Figure 4:**
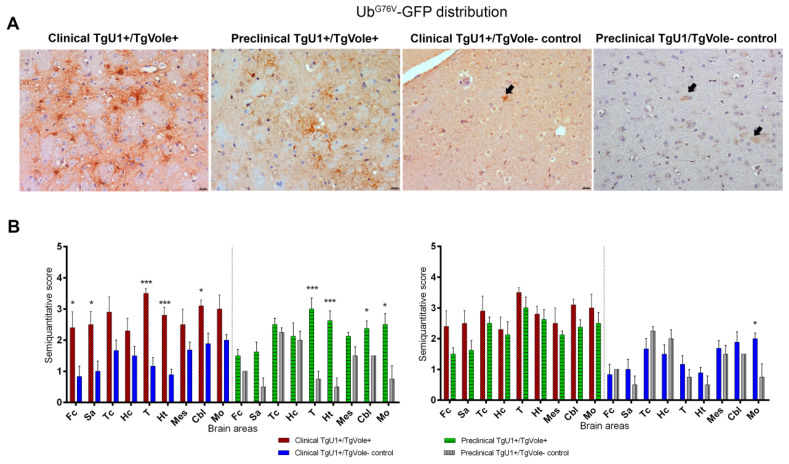
Ub^G76V^-GFP accumulation is more intense in clinical and preclinical TgU1^+^/TgVole^+^ mice than in healthy TgU1^+^/TgVole^−^ controls in certain brain areas. (**A**) Strong Ub^G76V^-GFP immunostaining was observed in the thalamus of clinical and preclinical TgU1^+^/TgVole^+^ mice, affecting cells whose morphology is compatible with reactive astrocytes. Healthy aged TgU1^+^/TgVole^−^ mice (clinical TgU1^+^/TgVole^−^ controls) showed granular immunostaining and filamentous ubiquitin aggregates in the neuropil (arrow). Healthy young TgU1^+^/TgVole^−^ mice (preclinical TgU1^+^/TgVole^−^ controls) showed slight intraneuronal Ub^G76V^-GFP immunolabeling (arrows). (**B**) Ub^G76V^-GFP distribution in the brains of clinical and preclinical TgU1^+^/TgVole^+^ mice and their age-matched controls. Ub^G76V^-GFP immunolabeling was semiquantitatively analyzed and scored on a scale of 0 (absence of immunolabeling) to 5 (very intense immunolabeling) in nine different brain areas. The number of animals within each group was: clinical TgU1^+^/TgVole^+^ *n* = 6 (4 female, 2 male), clinical TgU1^+^/TgVole^−^ controls *n* = 8 (6 female, 2 male), preclinical TgU1^+^/TgVole^+^ *n* = 5 (2 female, 3 male) and preclinical TgU1^+^/TgVole^−^ controls *n* = 5 (2 female, 3 male). Comparison of the Ub^G76V^-GFP immunolabeling profiles revealed significant differences between the group of clinical TgU1^+^/TgVole^+^ mice and clinical TgU1^+^/TgVole^−^ controls and between preclinical TgU1^+^/TgVole^+^ mice and preclinical TgU1^+^/TgVole^−^ controls in numerous brain areas. No differences were observed between clinical and preclinical TgU1^+^/TgVole^+^ mice. (* *p* < 0.05, *** p < 0.001, Mann-Whitney U test).

Ub^G76V^-GFP accumulation was very intense in the thalamus of both clinical and preclinical TgU1^+^/TgVole^+^ mice, indicating cellular UPS dysfunction in this brain area. Ubiquitinated deposits appeared as granular immunostaining in the neuropil and intense intracellular immunolabeling (Figure 4A). The morphology of the deposits found in both groups of TgU1^+^/TgVole^−^ controls was different from that observed in the clinical and preclinical TgU1^+^/TgVole^+^ groups. In clinical TgU1^+^/TgVole^−^ controls, we observed granular and filamentous Ub^G76V^-GFP in the neuropil, and in preclinical TgU1^+^/TgVole^−^ controls, we detected punctiform neuropil and intraneuronal immunostaining. The specificity of the Ub^G76V^-GFP immunolabeling was manifested by the absence of staining in clinically affected TgU1^−^/TgVole^+^ mice (Supplementary Figure S2).

When age-matched groups of mice were compared, a significantly increased Ub^G76V^-GFP accumulation was observed in the frontal cortex, septal area, thalamus, hypothalamus and cerebellum of clinical TgU1^+^/TgVole^+^ mice compared to their controls, the thalamus, again, being the most affected area. No significant differences were noticed in Ub^G76V^-GFP between clinical and preclinical TgU1^+^/TgVole^+^ mice, although clinical mice presented a higher accumulation of the GFP reporter in all brain areas. Preclinical mice showed, however, a significant upregulation of Ub^G76V^-GFP in the thalamus, hypothalamus, cerebellum and medulla oblongata compared to their controls. Clinical TgU1^+^/TgVole^−^ controls showed a more intense GFP immunolabeling in the medulla oblongata compared to preclinical TgU1^+^/TgVole^−^ controls, indicating that a certain degree of proteasomal dysfunction occurred due to aging in this area (Figure 4B).

**Figure 5:**
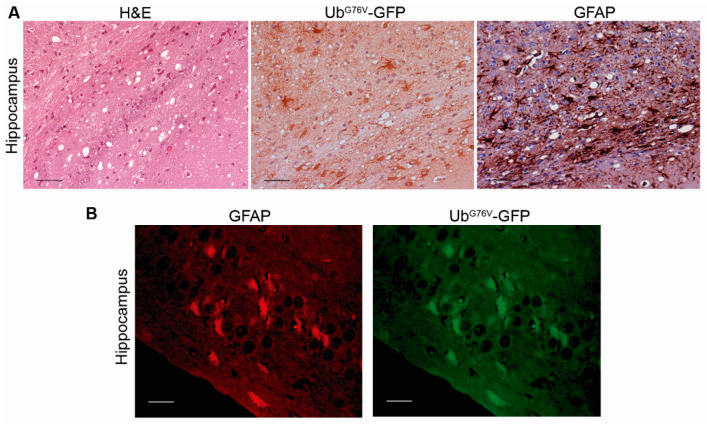
Ub^G76V^-GFP intracellular accumulation is observed in brain areas showing prion-associated neuropathology. (**A**) Hippocampus from a clinical TgU1^+^/TgVole^+^ mouse stained with hematoxylin and eosin and immunostained for Ub^G76V^-GFP and glial fibrillary acidic protein (GFAP). This animal shows severe spongiosis in the hippocampus and intense immunostaining for Ub^G76V^-GFP and GFAP. Strong Ub^G76V^-GFP immunolabeling is observed, affecting numerous cells that appear to be reactive astrocytes. (**B)** Dual immunofluorescence staining with anti-GFAP and anti-GFP antibodies revealed that numerous reactive astrocytes accumulated the Ub^G76V^-GFP reporter, suggesting that these cells can compensate for proteasome impairment and accumulate high amounts of ubiquitin conjugates before succumbing to the cytotoxic effect.

As aforementioned, we observed intracellular ubiquitinated deposition. The morphology of Ub^G76V^-GFP immunopositive cells in certain brain areas, such as the hippocampus and thalamus, was very similar to that of reactive astrocytes. This immunolabeling pattern for Ub^G76V^-GFP accumulation has already been described in RML-infected mice [34]. Therefore, we analyzed brain samples of spontaneously sick mice by immunohistochemistry and double immunofluorescence for GFAP (glial fibrillary acidic protein) and Ub^G76V^-GFP. GFAP staining revealed marked astrogliosis in certain brain areas. In these brain regions, we also observed intense Ub^G76V^-GFP immunolabeling affecting numerous cells whose morphology was consistent with hypertrophic astrocytes (Figure 5A). Dual immunofluorescence staining of Ub^G76V^-GFP and GFAP confirmed that numerous GFP-positive cells were reactive astrocytes (Figure 5B), indicating, as suggested before, that the UPS failure may regulate astrogliosis [44].

### 2.3. Correlation between BiP, PERK, PDI and Ub^G76V^-GFP Proteins and the Histopathological Features of the Disease

Spearman’s ρ correlation was calculated between the immunohistochemical scores for ER stress and UPS impairment markers to determine whether there is a relationship between them in the preclinical and clinical stages of the spontaneous prion disease. Spearman´s ρ was also calculated between spongiform lesions, PrP^Sc^ deposition and GFAP staining and immunohistochemical scores for ER stress and UPS impairment markers obtained in clinical mice, preclinical mice and their controls (Supplementary Figure S3) to identify a possible correlation between prion lesions, ER stress and UPS impairment. The correlation values and their statistical significance are presented in Table 1. In the preclinical stage of the disease, a positive correlation was found between protein scores for Ub^G76V^-GFP and PDI and between BiP and PDI. A positive correlation between these immunohistochemical markers was also detected in the clinical stage of the disease, in which we also observed that PERK and BiP were positively correlated. Interestingly, all immunohistochemical markers were positively correlated with spongiform lesions, PrP^Sc^ deposition and GFAP immunostaining in the clinical stage of the disease. All ER stress and UPS impairment markers were also positively correlated with spongiosis, PrP^Sc^ deposition and GFAP immunostaining in the preclinical stage of the disease, except for PERK, which was not correlated with PrP^Sc^ in the preclinical stage. These results suggest that there is a relationship between the accumulation of PERK, BiP, PDI and Ub^G76V^-GFP proteins and the neuropathological and neuroinflammatory phenomena that develop in the spontaneous prion disease. The level of correlation between spongiosis, PrP^Sc^ and GFAP is also displayed. Strong positive correlations were found between all these markers of prion disease in both the preclinical and clinical stages of the disease.

## 3. Discussion

The exact molecular mechanisms involved in prion-associated neurodegeneration are, at present, mostly unexplained. The unknowns are even greater in the case of spontaneous TSE, since their origin is still to be elucidated.

ER stress and UPS impairment have been suggested to play a pathogenic role in the neurodegenerative disorders associated with the accumulation of protein aggregates [45,46,47]. When ER proteostasis is disturbed due to the accumulation of misfolded proteins, cells experience ER stress, and the UPR survival pathway is initiated, leading to the upregulation of ER chaperones and foldases [16,48]. The UPR is vital in maintaining cell homeostasis. The UPR improves the ER-associated degradation (ERAD), and unnecessary proteins are eliminated via the UPS [16,29,49]. However, prolonged ER stress leads to cell death, and therefore, the accumulation of proteins could produce chronic UPR activation and subsequent neurodegeneration [50]. In addition, the accumulation of protein aggregates impairs the functionality of the UPS [51], and thus, the malfunction of this system has been extensively studied in the context of diseases produced by the deposition of aberrant proteins [46,52]. Moreover, it has been shown that misfolded PrP inhibits the proteasome [33,53] and that an impairment of the UPS occurs during the pathogenesis of acquired prion diseases [34]. Likewise, many other studies have shown an involvement of ER stress in infectious forms of TSE [5,7,17,54]. However, regarding familial prion diseases, the roles of ER stress and UPS dysfunction are more controversial [6,39,55].

In the present study, we evaluated the possible participation of ER stress and UPS impairment in the pathogenesis of spontaneous prion diseases. For this purpose, we analyzed the accumulation and brain distribution of PERK, BiP and PDI, proteins that are upregulated during ER stress [56]. We also evaluated the immunohistochemical accumulation of the Ub^G76V^-GFP reporter, which allows us to determine the functionality of the UPS in vivo [42] in the brains of TgU1^+^/TgVole^+^ mice. Although an upregulation of PDI and other disulfide isomerases such as Grp58/Erp57 has already been demonstrated in brain samples from sCJD patients [7,18,27], both ER stress and UPS impairment have not been deeply evaluated through the course of sporadic (putatively spontaneous) prion diseases. This is understandable, since sequent time-course studies are more complicated to perform in the context of spontaneous forms than in infectious TSE. In spontaneous TSE, it is difficult to establish the exact moment in which the PrP^Sc^ appears in the CNS and, therefore, the moment in which the neuropathogenic mechanisms begin. Thus, we selected brain samples obtained from TgU1^+^/TgVole^+^ mice culled at different ages in order to evaluate these pathogenic events in the preclinical and clinical stages of the spontaneous prion disease. The overexpression of the bank vole I109 PrP always leads to the development of spontaneous neurodegeneration in TgVole mice, making them a suitable model for the study of the spontaneous forms of TSE [40,41].

We performed an immunohistochemical approach to evaluate the accumulation of the ER stress markers BiP, PERK and PDI in the brains of clinical and preclinical TgU1^+^/TgVole^+^ mice and in age-matched controls. We observed positive immunolabeling for these markers in all groups of animals, regardless of age or genotype. Although several conditions, such as ER stress, can increase its expression, all these proteins play a role and are expressed in healthy cells [8].

Among these markers, PERK has received special attention in the field of neurodegenerative diseases. PERK is considered to be a strong mediator of neuronal dysfunction, since the PERK pathway initiates proapoptotic cascades [50], and it has been found to be increased in various neurodegenerative disorders. The upregulation and chronic activation of PERK has been reported in dopaminergic neurons from Parkinson’s patients, in hippocampal neurons in Alzheimer’s brains and in other tauopathies like Progressive Supranuclear Palsy (PSP) [57,58,59,60,61,62]. PERK has also been related to the decrease of the synthesis of synaptic proteins [63] and to the genetic risk for the onset of different tauopathies [62,64]. PERK activation in prion diseases, as detected by immunohistochemistry for phosphorylated PERK, has also been evaluated. Studies in the post-mortem brain tissue of human prion disease patients only detected activation of the PERK pathway in cases that had a concomitant tauopathy, suggesting that this mechanism was not a common feature of human prion pathogenesis [20,65]. In contrast, two studies from the same group demonstrated that oral treatment with a PERK inhibitor caused neuroprotection in prion diseased mice and in a mouse model of frontotemporal dementia, suggesting that PERK may be a very important therapeutic target against prion diseases or other neurodegenerative disorders [54,66]. A positive correlation of the levels of PERK with the extent of tau pathology has been also described in Alzheimer’s disease [58]. By estimating the expression levels of total PERK by immunohistochemistry, we observed a significant but discrete increase of this protein in the thalamus and hypothalamus of TgU1^+^/TgVole^+^ mice at the clinical stage. The upregulation of this protein was also observed in the medulla oblongata of preclinical mice. We also detected strong immunostaining for BiP in the areas where PERK appears overexpressed (Figure 2). Spearman´s test revealed a positive correlation between PERK and BiP levels in the clinical stage of the disease (Table 1). Therefore, the increase in the expression of BiP, considering that activation of the PERK pathway is related to BiP [13], may also produce the activation of PERK. BiP was strongly correlated with neuropathological changes in both the preclinical and clinical stages of the disease.

PERK, as BiP, appears to be correlated to prion neuropathological changes but not so strongly compared to the other markers (Table 1). The lack of prominent activation of the phosphorylated forms of PERK, elF2α and PKR in sporadic and other forms of human prion diseases [20] also suggests that this pathway may not play a crucial role in neuronal death in this type of prion disorder. However, these authors also suggested that the post-mortem dephosphorylation of these proteins could lead to a reduction in the detection of the activation of this pathway. When the same proteins were analyzed in samples from mice infected with sCJD, a slight activation of PERK was detected [20]. We observed similar results, considering that clinical TgU1^+^/TgVole^+^ mice showed an increase in PERK accumulation in a few brain areas when compared to TgU1^+^/TgVole^−^ controls.

Other pathways related to BiP/GRP78, such as IRE1, ATF6 or the Akt/PI3K pathway, could be of more importance, considering that an upregulation of the BiP protein and proteins of the PDI family have been reported several times in sporadic cases of CJD [7,18,27]. We observed that this may also be the case in our murine models of spontaneous prion disease. We detected significant differences in the accumulation of BiP and PDI in numerous brain areas of mice in the preclinical and clinical stages. In addition to that described for BiP, the Spearman’s test revealed a strong correlation between the upregulation of PDI and prion pathology in both the preclinical and clinical stages. This could be due to the fact that, in prion diseases, the activation of BiP produces a discrete activation of apoptotic pathways such as PERK but a strong activation of pro-survival mechanisms such as those regulated by IRE1 or ATF6 (not assessed in this study), which activate the expression of PDI [67]. Furthermore, the hypothesis that the IRE1 or ATF6 pro-survival pathways are activated instead of the PERK proapoptotic pathway in these diseases is reinforced by the increase in PDI observed in clinical TgU1^+^/TgVole^+^ mice. Both IRE1 and ATF6 activate pro-survival genes like BiP/GRP78 and 94 and protein disulfide isomerases (PDIs) [67]. Among the ER stress markers evaluated in this study, PERK is the one showing less significant differences between mouse groups and weaker Spearman´s positive correlations.

Whereas, as mentioned, the inhibition of PERK could represent a neuroprotective strategy, it has been shown that the reduction of BiP in vivo accelerates prion replication, suggesting that this chaperone plays an important role in preventing the propagation of PrP^Sc^ [68]. The increase of BiP levels has been demonstrated in cells infected with prions and in brains from patients affected by sCJD and vCJD [5,7]. An increase in the expression levels of BiP was observed in mice infected with the 139A scrapie strain. However, this increase was transitory, being only detected in the early stage of the disease [17]. Unlike this study, ours lacks data on ER markers in the brain of TgVole^+^ mice in the most initial stage of the clinical disease, because clinical TgU1^+^/TgVole^+^ mice were euthanized when they started showing severe signs of the prion disease. This makes it difficult to know if the levels of BiP were also incremented when the clinical signs started in these mice. Interestingly, we detected a significant increase of BiP levels in preclinical TgU1^+^/TgVole^+^ mice in multiple brain areas and a strong correlation with prion-associated pathology in the preclinical stage. These results may indicate that the upregulation of BiP starts earlier in the pathogenesis of the spontaneous disease developed by TgVole^+^ mice than in the scrapie-infected mice studied by Hetz and colleagues. We could attribute these discrepancies to the different transgenic lines used and to the different nature of the prion disease developed by the transgenic mice in both studies. However, we did not observe significant differences in BiP expression levels between clinical and preclinical mice, indicating, indeed, that BiP overexpression in our study could represent a response against the initial accumulation of PrP^Sc^. We can suggest that the upregulation of BiP starts early in the pathogenesis of the spontaneous prion disease developed by TgU1^+^/TgVole^+^ mice. The aforementioned positive Spearman´s correlations between BiP accumulation and neuropathological changes may suggest that, as the intensity of neuropathological changes in the brain increases, the level of accumulation of the BiP protein would also increase. This seems to disagree with the lack of significant differences in BiP accumulation between clinical and preclinical mice. Our study, however, is based on semiquantitative data, and further molecular and biochemical analyses are necessary to corroborate our results. However, although not significant, the accumulation of BiP was more intense in clinical than in preclinical TgU1^+^/TgVole^+^ mice in multiple brain areas (Figure 2). Additionally, Spearman’s correlations between BiP accumulation and the intensity of neuropathological changes were weaker in the clinical stage than in the preclinical stage (Table 1). The early upregulation of BiP and its positive correlation with prion neuropathology suggests that this chaperone may play a key protective role in the initial cellular response to prion infection.

Dynamic assays of the expression of PDI and other disulfide isomerases during scrapie experimental infection demonstrated that the upregulation of this protein begins at an early stage and continues to increase until terminal stage [6,17]. We observed that clinical TgU1^+^/TgVole^−^ controls show a significantly higher accumulation of PDI when compared with preclinical TgU1^+^/TgVole^−^ controls. The animals included in the control clinical group (clinical TgU1^+^/TgVole^−^ mice) were euthanized at coincidental ages to the animals of the clinical TgU1^+^/TgVole^+^ group to evaluate the immunohistochemical markers in age-matched animals. The average age of mice in both groups was, therefore, the same. The lifespan of laboratory mice is approximately two years [69]. Thus, these control mice are not considered old animals and are not a suitable model for the evaluation of the ER stress and UPS impairment related to aging.

What we consider of real importance is that clinical TgU1^+^/TgVole^+^ animals show significantly more intense accumulation of this protein than clinical controls in almost all brain areas analyzed. Preclinical TgU1^+^/TgVole^+^ mice also show significantly increased levels of PDI compared to their controls. As mentioned, we observed that the levels of PDI were strongly correlated to prion pathology in the preclinical and clinical stages of the disease. However, contrarily to what we observed with BiP, significant differences in PDI expression were observed between clinical and preclinical TgU1^+^/TgVole^+^ mice in many brain regions. These results indicate that spontaneous prion disease causes an upregulation of the PDI protein, which begins in the preclinical stage and continues until the terminal phase of the disease, correlating with the development of prion neuropathology, similarly to what has been previously described [6,17].

The upregulation of ER stress markers could represent a neuronal response against the accumulation of PrP^Sc^ and the development of spongiform lesions. Similar conclusions were obtained in previous studies in which an overexpression of ER stress markers was detected in the terminal phase of CJD-affected humans and murine scrapie models [7,18]. Among the ER stress markers studied here, PERK is the one showing the most discrete upregulation in mice affected by the spontaneous prion disease. We observed that the upregulation of BiP starts at the preclinical stage of the disease. The lack of significant differences in BiP deposition between the clinical and preclinical TgU1^+^/TgVole^+^ mice suggests that there is no significant increment of this protein during the clinical phase of the disease. On the contrary, PDI upregulation seems to start in the preclinical phase and continues increasing during the course of the disease. It should be noted, however, that we also observed an upregulation of PDI due to aging, since clinical TgU1^+^/TgVole^−^ controls accumulated significantly more PDI than preclinical TgU1^+^/TgVole^−^ controls.

Although we found some significant differences between spontaneously prion-affected and healthy control mice, we do not have enough evidence demonstrating that ER stress is an essential pathway during the course of the spontaneous neurodegenerative disorder developed by TgVole mice. Other studies have also shown that, although ER stress is apparently induced in prion diseases, the genetic ablation of proteins directly involved in the ER stress response or in ER-stress mediated apoptosis does not alter the progression of the disease in vivo [70,71]. Moreover, we found that PERK, BiP and PDI deposition were correlated with prion pathology, although PERK shows weaker Spearman´s positive correlations. (Table 1). These findings suggest that ER stress is produced during the pathogenesis of spontaneous prion diseases. However, further biochemical and molecular analyses are necessary to corroborate the role of ER stress in these neurodegenerative processes.

We also investigated the possible pathogenic role of proteasome impairment at different stages of the spontaneous prion disease in TgU1^+^/TgVole^+^ mice. Similar to ER stress markers, GFP immunoreactivity was detected in the brains of all mice expressing the Ub^G76V^-GFP reporter. However, in this case, the differences between the group of TgU1^+^/TgVole^+^ and age-matched controls were more evident in certain brain areas, such as the thalamus and hypothalamus (Figure 4). Spearman´s correlation test showed that Ub^G76V^-GFP deposition was strongly positively correlated with spongiform lesions and GFAP in both the preclinical and the clinical stages of the disease. We can suggest that, in the present study, as previously described in models of genetic prion diseases [39], UPS impairment is associated with the development of prion-associated neuropathology. Interestingly, we observed that, especially in the thalamic area, the morphology of the Ub^G76V^-GFP reporter deposits was very different between animals expressing the bank vole PrP (i.e., preclinical and clinical TgU1^+^/TgVole^+^ mice) and TgU1^+^/TgVole^−^ controls. This finding may be explained, because a high proportion of the cells in which the Ub^G76V^-GFP reporter was detected in clinical and preclinical TgU1^+^/TgVole^+^ mice appeared to be reactive astrocytes. This was later confirmed by dual immunofluorescence staining for GFAP and GFP (Figure 5). An intense accumulation of the Ub^G76V^-GFP reporter has already been described in the reactive astrocytes of prion-infected mice [33,34]. A proportional increase in the number of GFP-labeled astrocytes was observed as astrogliosis develops and, therefore, as the prion disease progresses [34]. We also found abundant GFP-positive astrocytes in certain brain areas of the preclinical TgU1^+^/TgVole^+^ mice, and a proportion of these cells was clearly increased in the same brain areas of clinical TgU1^+^/TgVole^+^ animals. It has been shown that depletion of the 26S proteasome causes astrogliosis, manifested by an increase in GFAP and vimentin expression [44]. We also detected strong correlations between the accumulation of Ub^G76V^-GFP and GFAP immunostaining. By contrast, other in vitro experiments have found that proteasome inhibition rather produces a decrease in the transcript levels of GFAP [72]. However, numerous studies agree that astrocytes are more resistant than neurons to the toxic effects of proteasome malfunction [72,73,74,75], which has been attributed to their high expression of heat shock proteins [73,76]. Thus, we can suggest, as has been done previously [34], that astrocytes have other routes to compensate for UPS dysfunction during the course of prion diseases. Therefore, they would be able to accumulate the Ub^G76V^-GFP reporter for longer than neurons before suffering the toxic effects of proteasome impairment.

In TgU1^+^/TgVole^−^ controls, we detected filamentous and granular GFP-labeled aggregates in the neuropil, but we did not observe the astrocytic pattern found in TgU1^+^/TgVole^+^ mice (Figure 4A). In addition to that described above, it has been shown that misfolded proteins produce an astrocytic upregulation of UPS proteins and proteasome impairment [77]. This phenomenon could explain the morphological differences in Ub^G76V^-GFP deposits between TgU1^+^/TgVole^+^ and TgU1^+^/TgVole^−^ mice.

We observed that the thalamus seems to be more affected by these mechanisms than other brain regions. The differential responses to ER stress and proteasome impairment shown by different brain areas have been reported in several studies, but no clear explanation has been given for these findings [17,62]. These differences might be due to the fact that the different cell populations of each brain region respond differently to the pathogenic phenomena occurring in prion diseases, as it has been demonstrated with neuroinflammation [78].

As it has been previously mentioned, the molecular mechanisms on how the conversion of PrP^C^ in PrP^Sc^ starts are still unknown. Even though it is not known what triggers this initial conversion in spontaneous prion diseases, as in other types of TSE, one of the causes of pathogenesis is that the intracellular accumulation of PrP^Sc^ leads to cytotoxicity [33]. Based on the results of this study, the proteasome impairment could be preventing the degradation of aggregated proteins, causing the accumulation of protein aggregates and impairment of the functional capacity of the UPS, contributing to the pathogenesis of spontaneous TSE as it has been reported in other kinds of prion diseases [33,34].

Despite these results being an approach on how the studied mechanisms affect spontaneous TSE, they are not sufficient to clearly demonstrate that ER stress or UPS dysfunction are major role players in the pathogenesis of spontaneous prion diseases. Although certain in vivo studies have positively demonstrated that both the upregulation of PDI and UPS impairment are mediators of prion pathogenesis during the course of infectious TSE [6,33,34], others have failed to show a significant contribution of ER stress or proteasome malfunction in prion pathology [20,39]. It should be noted, however, that most of the studies about ER stress and UPS impairment throughout the course of the disease (both preclinical and clinical stages) have focused on acquired forms of TSE, using animals inoculated to develop the disease [6,17,34]. In this study, however, we assessed the role of ER stress in spontaneous prion diseases, both in the preclinical and clinical stages. Although there is not a clear resolution to this problem, we suggested that spontaneous, acquired and genetic prion diseases should be treated as different pathologies in terms of pathogenesis. We believe that the different results obtained by other groups can be explained by the differences in the pathogenesis between these different types of prion diseases [34,39].

We cannot know for certain if, during the spontaneous TSE of TgVole^+^ mice, the ER stress and the UPS impairment are mere secondary events associated with prion neuropathology, as previously suggested [18,39], or whether the generated PrP^Sc^ in our case does not experience an ER metabolism but a Golgi-based quality control [6,79], and therefore, it does not activate the UPR and, consequently, the mechanisms of ER-associated degradation. Nevertheless, as already mentioned, further molecular and biochemical studies are necessary to confirm the participation of ER stress and proteasome malfunction in the pathogenesis of spontaneous prion diseases. Likewise, the role of other mechanisms of proteostasis regulation, as well as the role of other ER resident chaperones involved in folding quality control, should be also explored in relation to the pathogenesis of these diseases. Regarding the results observed in this study, we consider that the IRE1 and ATF6 pathways could be also involved in sporadic prion diseases. In further studies, we will assess the roles of these molecules in the same model of spontaneous prion diseases pathogenesis, and we will also perform biochemical analyses for the already studied proteins.

## 4. Materials and Methods

### 4.1. Mice

To study the accumulation and brain distribution of the ER stress markers PERK, BiP and PDI and the proteasome function reporter Ub^G76V^-GFP over the course of spontaneous prion diseases, we selected mice both expressing ~3–4× the I109 polymorphic variant of bank vole PrP and Ub^G76V^-GFP (TgU1^+^/TgVole^+^ mice). This model was generated by breeding mice overexpressing bank vole PrP (TgVole mice) [41,80], with the Ub^G76V^-GFP/1 mouse line [42] (TgU1 mice). The overexpression of bank vole PrP leads to the development of a spontaneous prion disease that, in this particular transgenic line, clinically appears at ~180–200 days of age and resembles a transmissible prion disease described in other transgenic lines overexpressing the same protein [40]. The co-expression of the Ub^G76V^-GFP allows the study of the impairment of the UPS in a model of spontaneous prion disease, since the accumulation of the Ub^G76V^-GFP reporter indicates a deterioration of this system [42]. Prior to analyzing the data, we corroborated that the co-expression of Ub^G76V^-GFP had no apparent effect in the onset of the spontaneous prion disease of the TgVole model. TgU1^+^/TgVole^+^ mice succumbed to the spontaneous neurodegenerative disorder at ages similar to those observed in TgVole animals (~180–200 days of age), with no significant differences between their survival periods (Supplementary Figure S4).

We also aimed at studying ER stress and UPS impairment at different stages of the disease. Thus, we analyzed the accumulation of ER stress markers and Ub^G76V^-GFP in TgU1^+^/TgVole^+^ mice that developed the spontaneous prion disorder (TgU1^+^/TgVole^+^ mice) and in TgU1^+^/TgVole^+^ mice that were euthanized at ~60 days of age before the onset of the disease (preclinical TgU1^+^/TgVole^+^ mice). Considering that aging produces oxidative stress, which can decrease both the UPS activity and the functionality of ER chaperones [81,82], we selected two groups of age-matched TgU1^+^/TgVole^−^ mice that were used as controls (clinical TgU1^+^/TgVole^−^ controls and preclinical TgU1^+^/TgVole^−^ controls, respectively). Since TgU1^+^/TgVole^−^ mice do not express TgVole PrP, they do not develop the spontaneous prion disease. Therefore, four groups of mice were included in this study, clinical TgU1^+^/TgVole^+^ (including six animals: four females and two males, ~180–200 days of age), clinical TgU1^+^/TgVole^−^ controls (with eight mice: six females and two males, aged ~180–200), preclinical TgU1^+^/TgVole^+^ (with five animals: two females and three males, ~60 days of age) and preclinical TgU1^+^/TgVole^−^ controls (including five mice: two females and three males, aged ~60 days).

In addition, to evaluate whether the expression of the Ub^G76V^-GFP reporter had any interference with PERK, BiP or PDI deposition, several brain samples from TgVole mice (hereafter encoded as TgU1^−^/TgVole^+^ mice) were used for the immunostaining of these proteins.

Mice were monitored on a daily basis for the onset of neurologic signs. Animals of the clinical group were humanely euthanized by CO_2_ asphyxiation followed by cervical dislocation upon the detection of severe clinical signs of disease (i.e., locomotor disorders, poor body condition and any signs of impaired feeding ability) and their brains collected. Preclinical and control mice were euthanized following the same procedure at corresponding time points (i.e., ~60 and ~180–200 days of age). No unexpected deaths were observed during the course of the study. No significant signs of distress or pain were observed in the animals other than those related to the onset of severe neurological signs and the moment in which the mice were euthanized.

### 4.2. Histological and Immunohistochemical Analyses

Sections from paraffin-embedded mouse brains (4-μm-thick) were cut and collected on glass slides and dried at 56 °C for 24 h.

Brain sections (1 per mouse) were stained with hematoxylin and eosin to visualize spongiform lesions if present.

For the detection of BiP and PERK proteins, a manual immunohistochemical technique was performed. Antigen retrieval was done with a citrate buffer (pH 6.0) for 10 min at 96 °C, and endogenous peroxidase activity was blocked using a blocking reagent (Dako, Glostrup, Denmark) for 15 min. Next, sections were incubated with antibodies (both at 1:200 dilution) against the phosphorylated form of PERK (ab79483, Abcam, Cambridge, United Kingdom) and GRP78/BiP (ab108613, Abcam, Cambridge, United Kingdom) overnight at 4 °C. Then, samples were incubated with an enzyme-conjugated anti-rabbit Envision polymer (Dako, Glostrup, Denmark) for 30 min at room temperature, and diaminobenzidine (DAB, Dako, Glostrup, Denmark) was used as the chromogen. The specificity of the immunohistochemical technique for the BiP/Grp78 and PERK proteins was determined by the absence of immunostaining in mouse brain sections in which the primary antibody incubation was omitted.

PDI, GFAP (glial fibrillary acidic protein) and PrP^Sc^ immunostaining were performed using an automated immunostaining system (Dako Autostainer, Dako, Glostrup, Denmark), similar to what has been previously described [83]. After deparaffination and rehydration, sections intended for PrP^Sc^ immunostaining were pretreated with formic acid 98% and proteinase K (4 µg/mL; Roche, Basel, Switzerland) prior to hydrated autoclaving for 10 min at 121 °C, while the samples used for PDI immunostaining were only subjected to the heat treatment. Next, endogenous peroxidase activity was blocked using a blocking reagent (Dako, Glostrup, Denmark), and the samples were incubated for 1 h at room temperature with primary antibodies: anti-PrP 6H4 antibody (1:100; Prionics, Zurich, Switzerland) or anti-PDI antibody (1:200; sc-166474, Santa Cruz Biotechnology, Dallas, Texas, USA). Sections were then incubated with an enzyme-conjugated anti-mouse Envision polymer (Dako, Glostrup, Denmark) followed by diaminobenzidine (DAB, Dako, Glostrup, Denmark), which was used as the chromogen. The specificity of the immunohistochemical technique for the PDI and GFAP proteins was determined by the lack of immunostaining in mouse brain samples in which the primary antibody was omitted. Brain samples from noninoculated mice were used as controls for PrP^Sc^ accumulation and resulted negative for PrP^Sc^ immunostaining.

Although Ub^G76V^-GFP/1 mice expressed a GFP-tagged ubiquitin, which, therefore, possesses autofluorescence, we observed that such fluorescence was weak and, therefore, difficult to evaluate. We decided to perform an immunohistochemical technique to detect the GFP protein increasing its detection, a strategy previously done in multiple studies using this transgenic line [34,39,84]. GFP immunostaining was performed as described elsewhere [33], with a few modifications. Briefly, sections were incubated with a peroxidase-blocking reagent (Dako, Glostrup, Denmark) for 30 min, followed by 30 min with 10% goat serum in PBS (phosphate-buffered saline). Immunodetection was performed overnight at 4 °C using a rabbit polyclonal anti-GFP primary antibody (1:2500; anti-GFP antibody-ChIP Grade, ab290, Abcam, Cambridge, United Kingdom). The anti-rabbit Envision polymer (Dako, Glostrup, Denmark) was used as the visualization system and DAB as the chromogen. The specificity of the Ub^G76V^-GFP immunolabeling was determined by the absence of staining in TgU1^−^/TgVole^+^ controls.

Brain sections were examined using a Zeiss Axioskop 40 optical microscope (Zeiss, Oberkochen, Germany). BiP, PERK, PDI and GFP immunostaining were blindly evaluated in 9 encephalic areas: frontal cortex (Fc), septal area (Sa), cerebral cortex at the level of the thalamus (Tc), hippocampus (Hc), thalamus (T), hypothalamus (Ht), mesencephalon (Mes), cerebellum (Cbl) and medulla oblongata (Mo) (42) and semiquantitatively scored on a scale of 0 (absence of immunolabeling) to 5 (very intense immunolabeling).

### 4.3. Immunofluorescence Staining

Immunofluorescence staining of paraffin-embedded brain sections was performed as described previously [85]. Tissues were deparaffinated, rehydrated and, subsequently, blocked using 1% H_2_O_2_ for 30 min. Sections were then pretreated with 0.1% Triton X-100 for 3 h at room temperature and then subjected to hydrated autoclaving (121 °C, 10 min). Immunodetection was performed overnight using primary antibodies: anti-GFP (1:200; Abcam, Cambridge, United Kingdom) and anti-GFAP (1:200; Dako, Glostrup, Denmark), which were diluted in a solution of 0.1% Triton X-100. Sections were then washed in cold PBS and incubated with secondary antibodies: goat anti-mouse IgG biotin conjugate (1:100; Invitrogen, Carlsbad, California, USA) and Alexa Fluor 594 streptavidin conjugate (1:1000; Invitrogen, Carlsbad, California, USA) for 1 h in darkness. Finally, slides were washed and mounted in aqueous medium.

Sections were analyzed using a Zeiss fluorescence microscope Axioskop HBO (Carl Zeiss MicroImaging, Oberkochen, Germany).

### 4.4. Data Analysis

Significant differences of PERK, BiP, PDI and Ub^G76V^-GFP immunostaining between the experimental groups of mice were evaluated using a two-sided Mann Whitney U test and considered significant at *p* < 0.05. Correlations between the immunostaining of ER stress markers, Ub^G76V^-GFP and histopathological lesions were determined using the nonparametric Spearman´s rank correlation coefficient (*p* < 0.05).

All data analyses and graphs were performed using GraphPad Prism version 6.0 (GraphPad Software, La Jolla, California, USA).

This study is based on semiquantitative immunohistochemical data, and further molecular and biochemical analyses will be performed to obtain quantitative measures that allow us to obtain more solid conclusions on ER stress and UPS impairment pathogenic mechanisms in spontaneous prion diseases.

### 4.5. Ethics Statement

TgVole mice were obtained from the breeding colony at CIC bioGUNE (Spain) and were bred with TgU1 mice (expressing Ub^G76V^-GFP reporter) at the same facility. All procedures involving animals adhered to the guidelines included in the Spanish law “Real Decreto 1201/2005 de 10 de Octubre” on the protection of animals used for experimentation and other scientific purposes, which is based on the European Directive 86/609/EEC on Laboratory Animal Protection. The project was approved by the Ethical Committee on Animal Welfare (project code assigned by the Ethical Committee P-CBG-CBBA-1413) and performed under their supervision.

## Figures and Tables

**Table 1:** Spearman’s correlation values between scores of endoplasmic reticulum (ER) stress and proteasome impairment and prion-associated histopathological lesions. Correlations were estimated using the full set of data obtained in all brain areas. n.s.: no statistically significant value. Spearman’s correlation * *p* < 0.05, ** *p* < 0.01, *** *p* < 0.001 and **** *p* < 0.0001.

		Ubiquitin	PDI	PERK	BiP	Spongiosis	PrP^Sc^	GFAP
Preclinical stage (Preclinical +controls)	Ubiquitin	----	0.2676 *	0.1249 ^n.s.^	0.1950 ^n.s.^	0.4701 ****	0.6190 ****	0.6020 ****
	PDI	0.2676 *	----	−0.2014 ^n.s.^	0.4944 ****	0.4183 ***	0.5211 ****	0.4302 ***
	PERK	0.1249 ^n.s.^	−0.2014 ^n.s.^	----	0.1496 ^n.s.^	0.2894 *	0.2041 ^n.s.^	0.2518 *
	BiP	0.1950 ^n.s.^	0.4944 ****	0.1496 ^n.s.^	----	0.4008 ***	0.5105 ****	0.5214 ****
	Spongiosis	0.4701 ****	0.4183 ***	0.2894 *	0.4008 ***	----	0.7603 ****	0.6152 ****
	PrP^Sc^	0.6190 ****	0.5211 ****	0.2041 ^n.s.^	0.5105 ****	0.7603 ****	----	0.8550 ****
	GFAP	0.6020 ****	0.4302 ***	0.2518 *	0.5214 ****	0.6152 ****	0.8550 ****	----
Clinical stage (Clinical +controls)	Ubiquitin	----	0.3131 ***	0.1124 ^n.s.^	0.1606 ^n.s.^	0.4616 ****	0.5947 ****	0.5059 ****
	PDI	0.3131 ***	----	0.1962 ^n.s.^	0.4457 ****	0.4007 ****	0.3172 **	0.4170 ****
	PERK	0.1124 ^n.s.^	0.1962 ^n.s.^	----	0.2819 **	0.2554 *	0.3182 **	0.2663 *
	BiP	0.1606 ^n.s.^	0.4457 ****	0.2819 **	----	0.2573 *	0.3391 ***	0.5395 ****
	Spongiosis	0.4616 ****	0.4007 ****	0.2554 *	0.2573 *	----	0.8507 ****	0.6619 ****
	PrP^Sc^	0.5947 ****	0.3172 **	0.3182 **	0.3391 ***	0.8507 ****	----	0.7958 ****
	GFAP	0.5059 ****	0.4170 ****	0.2663 *	0.5395 ****	0.6619 ****	0.7958 ****	----

PDI: Protein disulfide isomerase; PERK: PKR-like endoplasmic reticulum kinase; BiP: Binding immunoglobulin protein; PrP^Sc^: Pathological prion protein; GFAP: Glial fibrillary acidic protein.

## Data Availability

The data presented in this study are available within the article text, figures and supplementary materials.

## Supplementary Materials

The following are available online at https://www.mdpi.com/1422-0067/22/1/465/s1: Figure S1: The expression of Ub^G76V^-GFP does not alter the immunohistochemical distribution or intensity of the ER stress markers. Figure S2: Ub^G76V^-GFP immunostaining in the thalamic area of a clinical TgU1^+^/TgVole^+^, mouse, a clinical TgU1^+^/TgVole^-^ control and a clinical TgU1^-^/TgVole^+^ mouse. Figure S3: Spongiosis, PrP^Sc^ and GFAP deposition profiles of clinical and preclinical TgU1^+^/TgVole^+^ mice and their TgU1^+^/TgVole^-^ controls in the 9 studied CNS areas. Figure S4: The co-expression of the Ub^G76V^-GFP does not affect the onset and characteristics of the spontaneous prion disease of the TgVole model

## Abbreviations

ATF6	Activating transcription factor 6
BiP/Grp78	Binding immunoglobulin protein
CBL	Cerebellum
CNS	Central Nervous System
DAB	Diaminobenzidine
ER	Endoplasmic Reticulum
ERAD	Endoplasmic Reticulum Associated Degradation
FC	Frontal Cortex
GFAP	Glial fibrillary acidic protein
Hc	Hippocampus
Ht	Hypothalamus
IRE1	Inositol-requiring enzyme 1
Mes	Mesencephalon
Mo	Medulla oblongata
PERK	PKR-like endoplasmic reticulum kinase
PDI	Protein disulfide isomerase
PrP^Sc^	Pathological prion protein
PrP^C^	Cellular prion protein
Sa	Septal Area
PSP	Progressive Supranuclear Palsy
sCJD	Sporadic Creutzfeldt-Jakob Disease
T	Thalamus
TC	Cortex at the level of the thalamus
Tg	Transgenic
TSE	Transmissible Spongiform Encephalopathies
Ub	Ubiquitin
UPR	Unfolded Protein Response
UPS	Ubiquitin-Proteasome System
vCJD	Variant Creutzfeldt-Jakob Disease
