# Supplementary figures and images for: Prion-Associated Neurodegeneration Causes Both Endoplasmic Reticulum Stress and Proteasome Impairment in a Murine Model of Spontaneous Disease
Source: Int J Mol Sci. 2021 Jan 5;22(1):465. doi: 10.3390/ijms22010465 (PMC7796520; doi:10.3390/ijms22010465)

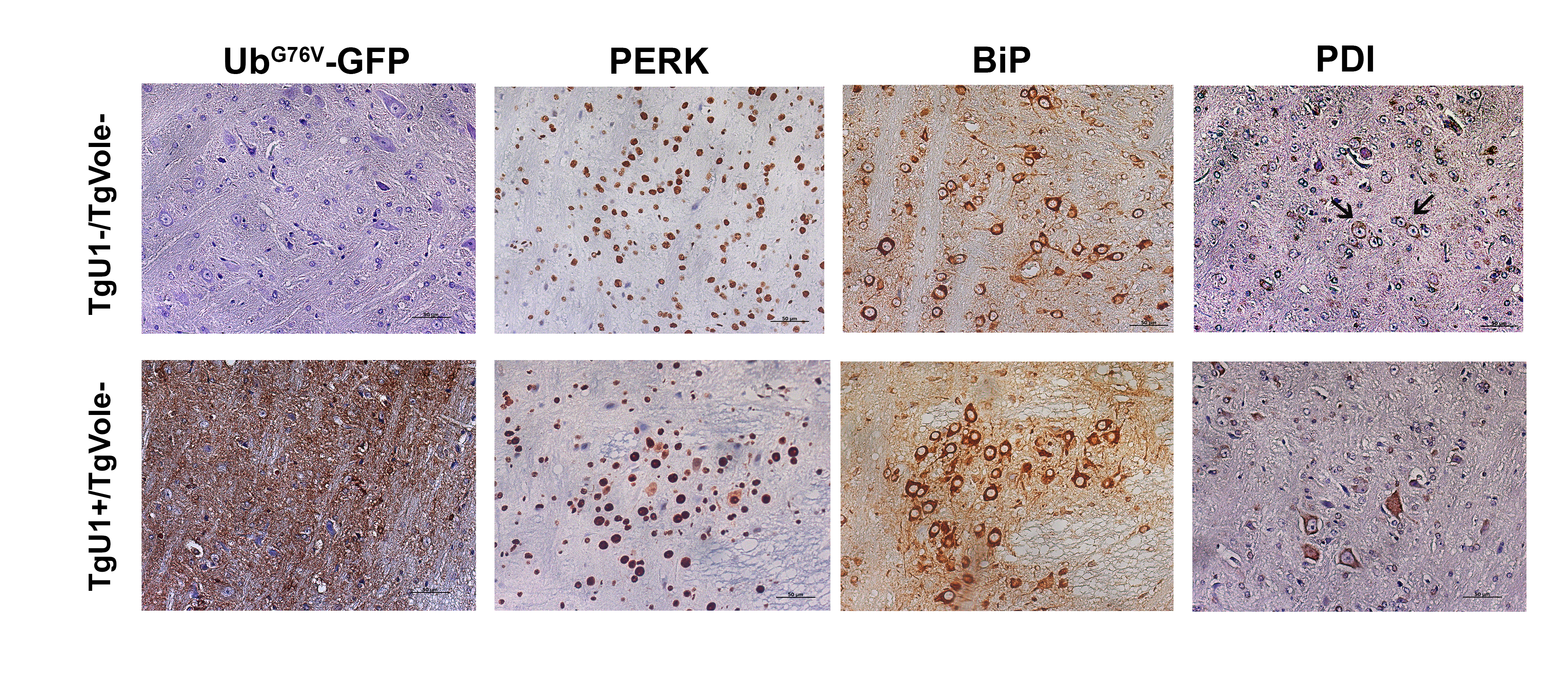

Supplement: Supplementary file 1 [file ijms-22-00465-s001.zip › Supplementary Figure 1.tif]

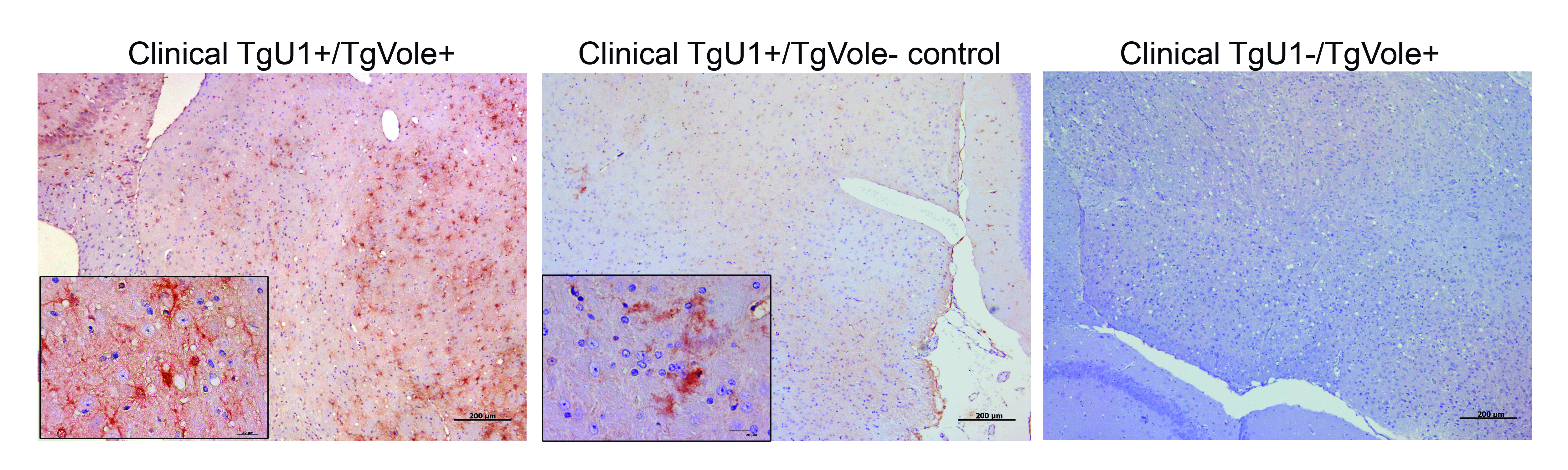

Supplement: Supplementary file 1 [file ijms-22-00465-s001.zip › Supplementary Figure 2.tif]

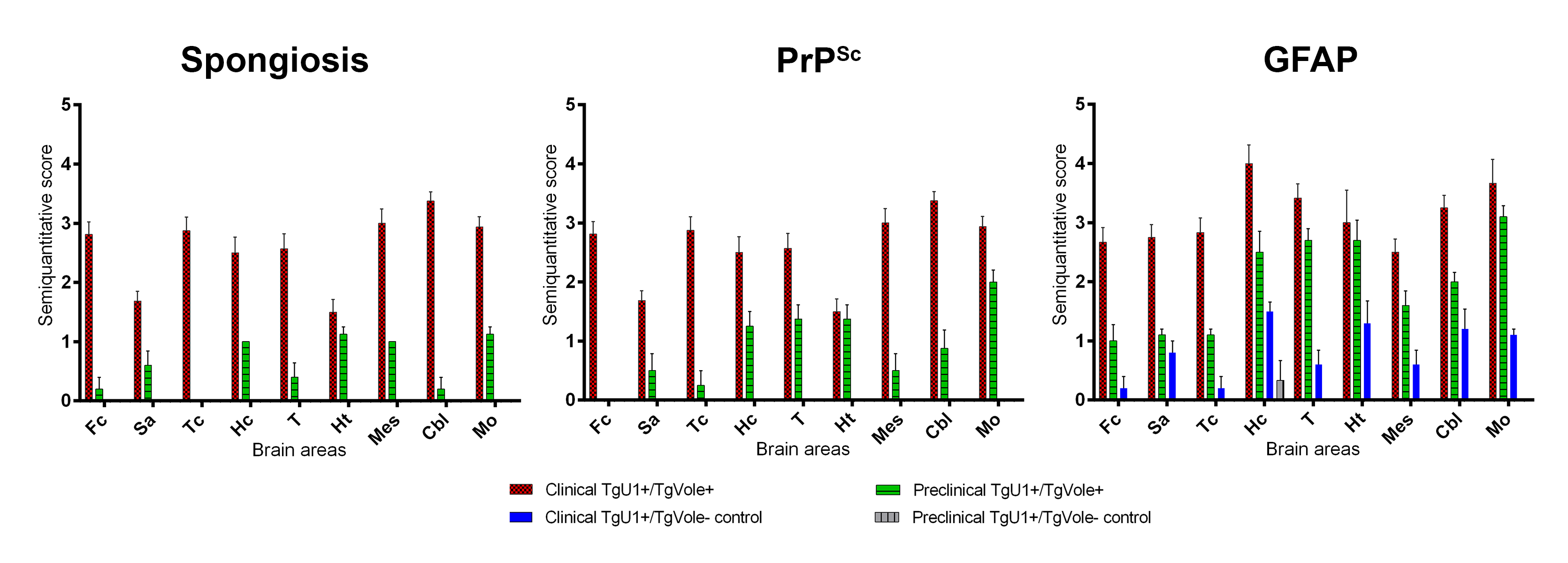

Supplement: Supplementary file 1 [file ijms-22-00465-s001.zip › Supplementary Figure 3.tif]

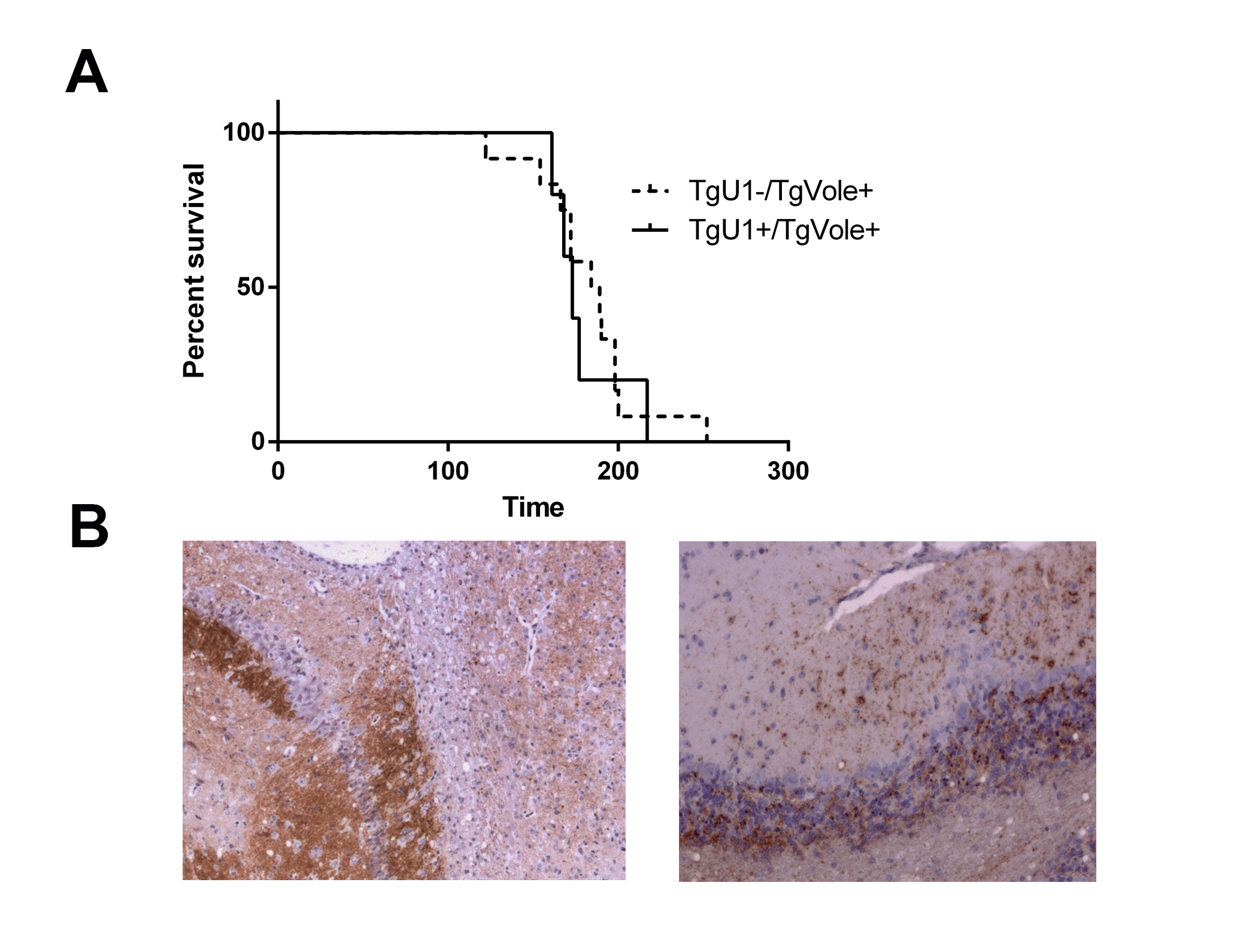

Supplement: Supplementary file 1 [file ijms-22-00465-s001.zip › Supplementary Figure 4.tif]
